# Re-evaluating the cost and cost-effectiveness of rotavirus vaccination in Bangladesh, Ghana, and Malawi: A comparison of three rotavirus vaccines

**DOI:** 10.1016/j.vaccine.2018.10.068

**Published:** 2018-11-26

**Authors:** Clint Pecenka, Frederic Debellut, Naor Bar-Zeev, Palwasha Anwari, Justice Nonvignon, Md Shamsuzzaman, Andrew Clark

**Affiliations:** a PATH, 2201 Westlake Ave, Suite 200, Seattle, WA 98121, USA; b PATH, Rue de Varembé 7, 1202 Geneva, Switzerland; c International Vaccine Access Center, Department of International Health, Johns Hopkins Bloomberg School of Public Health, 615 N. Wolfe Street, Baltimore, MD 21205, USA; d Malawi-Liverpool-Wellcome Trust Clinical Research Programme, College of Medicine, University of Malawi, Blantyre, Malawi; e Afghanistan National Immunization Technical Advisory Group, District 10, Kabul, Afghanistan; f School of Public Health, University of Ghana, Legon, Ghana; g National Immunization Program of Bangladesh, Directorate General of Health Services (DGHS), EPI Bhaban, Mohakhali, Dhaka 1212, Bangladesh; h London School of Hygiene and Tropical Medicine, Keppel Street, London WC1E 7HT, United Kingdom

**Keywords:** Rotavirus vaccines, Cost-effectiveness, New vaccine introduction, Gavi countries

## Abstract

**Introduction:**

Diarrhea is a leading cause of mortality worldwide and rotavirus accounts for many of these deaths. As of August 2018, 96 countries have introduced rotavirus vaccines into their immunization programs. Two rotavirus vaccines, Rotarix® and RotaTeq®, have been WHO-prequalified since 2009, with Rotarix® being the preferred product of most Gavi-supported countries. ROTAVAC® and ROTASIIL® have both been prequalified recently.

**Materials and methods:**

We reevaluated the costs and cost-effectiveness of rotavirus vaccination in Bangladesh, Ghana, and Malawi and compared Rotarix®, ROTAVAC®, and ROTASIIL® in each country. For consistency with previously published analyses in these countries, we used the same Excel-based cohort model and much of the same data as the original analyses. We varied the expected price (with and without Gavi subsidy), wastage, and incremental health system costs associated with each vaccine. We assumed the same efficacy and waning assumptions following administration of two or three doses for the respective product.

**Results:**

The discounted cost per DALY averted compared to no vaccination ranged from 0.3 to 1.3 times GNI per capita for each vaccine. With the Gavi subsidy, the average cost-effectiveness ratios were below 0.3 times GNI per capita in all three countries. Though critical empirical cost data are not yet available, Rotarix® is the least costly and most cost-effective product in the countries examined in this modelling study. However, small decreases in the incremental health system cost for other products could result in cost and cost-effectiveness outcomes that match or surpass those of Rotarix®.

**Conclusion:**

Countries may wish to consider new rotavirus vaccines entering the market. Countries should carefully examine multiple product attributes including price and the incremental health system costs associated with each vaccine. These costs will vary by country and may be a defining factor in determining the least costly and most cost-effective product for the population.

## 1 Introduction

Diarrhea is a leading cause of childhood mortality worldwide, causing 9% of all under-5 deaths [1], [2], [3]. Rotavirus accounts for 24–37% of these deaths [3], resulting in an estimated 200,000 deaths annually [4]. The World Health Organization (WHO) recommended rotavirus vaccination in all countries’ national immunization programs in 2009, and as of August 2018 96 countries have introduced rotavirus vaccine [5], [6]. Gavi, the Vaccine Alliance provides support for rotavirus vaccination to eligible low- and middle-income countries (LMICs), and more than 40 countries have introduced with Gavi support [6], [7]. Numerous other countries have recently been approved for Gavi rotavirus vaccine support [8]. Recent approvals in countries with large birth cohorts include Afghanistan, Bangladesh, Democratic Republic of the Congo, Nigeria, and Pakistan. India has introduced rotavirus vaccination in at least six states and will continue to expand the program nationally with domestically manufactured products (ROTAVAC® and ROTASIIL®). Together, these six countries account for more than 30% of the world’s infants [9].

The introduction of rotavirus vaccination in these countries promises to substantially reduce rotavirus disease burden and deaths but may pressure current vaccine supplies. Two rotavirus vaccines have been WHO-prequalified for global use since 2009: Rotarix® (a registered trademark of GlaxoSmithKline Biologicals SA, used under license by GlaxoSmithKline Inc.) and RotaTeq® (a registered trademark of Merck & Co., Inc.) [10]. However, ROTAVAC® (Bharat Biotech International Limited) and ROTASIIL® (Serum Institute of India) were recently prequalified by WHO [11], [12]. WHO-prequalification ensures a product meets quality, safety and efficacy requirements allowing its purchase by international procurement agencies. This enables more manufacturers to enter the international market, increasing choice, and lowering supply pressure.

Product selection is a complex, multifactorial decision, and each introducing country assesses the advantages and disadvantages of competing products. In addition to differences in efficacy and safety among products, decision-makers may consider the potential costs of introduction and implementation and how this will affect cost-effectiveness. Such costs include the vaccine costs (e.g., price of the vaccine, international handling, transportation costs, and wastage) and costs to the health system (e.g., subnational vaccine distribution, cold chain expansion, and staff training and labor for service delivery or program management). As many countries transition away from Gavi financial support and toward self-financing of vaccines, these economic considerations become increasingly important.

In this analysis, we reevaluate cost-effectiveness studies previously conducted in Bangladesh, Ghana, and Malawi [13], [14], [15]. Our objective is to compare the economic impacts of three vaccines (Rotarix®, ROTAVAC®, and ROTASIIL®) as though they were all available at the time of the original analyses. Although RotaTeq® was an available product at the time, the previous studies each examined Rotarix® as a country-preferred product. As a result, we did not include RotaTeq® in this analysis. Using previously published national cost-effectiveness studies allows us to leverage model inputs that have already been gathered, reviewed, and accepted by participating national stakeholders. Our aim is not to advocate for any particular product, but to explore the sensitivity of costs and cost-effectiveness of available products to a range of transparent assumptions within and across three countries at different levels of Gavi support.

## 2 Materials and methods

### 2.1 Model inputs and assumptions

The authors recently undertook impact and cost-effectiveness analyses of rotavirus vaccination in Bangladesh, Ghana, and Malawi [13], [14], [15]. Each analysis used the TRIVAC model, a static cohort model developed at the London School of Hygiene and Tropical Medicine with support from the Pan American Health Organization’s ProVac Initiative. TRIVAC models consecutive birth cohorts to generate impact and cost-effectiveness estimates of rotavirus and other vaccines. Model inputs include demographic projections, disease incidence and mortality rates, vaccine efficacy (allowing for possible waning), vaccine coverage, timeliness of uptake, vaccine program costs, health service utilization rates, and treatment and households costs [16]. Analyses were adapted to country context and undertaken at different time points relative to vaccine introduction, which had implications for the use of local data in the analysis. For example, the Bangladesh analysis was undertaken prior to vaccine introduction and used projections for vaccine effectiveness. Conversely, the Malawi analysis was undertaken after vaccine introduction and local effectiveness data were available to inform the analysis. All examined Rotarix® as a country-preferred product.

The current analysis follows the prior studies as closely as possible and uses the same assumptions and data wherever possible, only varying inputs to reflect core characteristics of the new products that differ from Rotarix®. We do not attempt to harmonize inputs to make the results comparable across countries. Rather, we maintain consistency with the previously published studies to allow comparisons between vaccine products in the individual countries. Some parameters (e.g. severity of disease or treatment seeking rates) may differ across countries. We do not explore these cross country differences here but the rationale for those input parameters can be found in the original papers. Tables 1 and S1 in supplementary materials highlight the characteristics and data inputs that remain consistent between the initial studies and this analysis.

**Table 1:** Key model inputs consistent with prior analyses.

Parameter	Bangladesh	Ghana	Malawi	Source/s
**Annual rotavirus events pre-vaccination**				
Non-severe cases/100,000 aged <5 years	7300	9290	9201	Bangladesh: assumption based on [27], [28], [29] Ghana: assumption based on [30], [31] Malawi: assumption based on [16], [32]
Severe cases/100,000 aged <5 years	2700	710	799	
Rotavirus gastroenteritis mortality/100,000 aged <5 years	12.42	46.15	33.48	Bangladesh: assumption based on [4], [33] Ghana: calibrated to align with [4] Malawi: assumption based on [34], [35], [36]
Non-severe outpatient visits/100,000 aged <5 years	3431	4181	5797	Bangladesh: assumption based on [37], [38] Ghana: [39] Malawi: [15]
Severe outpatient visits/100,000 aged <5 years	1269	320	503	Bangladesh: assumption based on [37], [38] Ghana: [39] Malawi: [15]
Hospitalizations/100,000 aged <5 years	1107	568	503	Bangladesh: assumption based on [26], [27] Ghana: assumption based on local expert opinion Malawi: [15]

**Disability weight for DALY calculations**				
Rotavirus (non-severe) cases	0.188	0.188	0.202	Bangladesh and Ghana [40] Malawi [41]
Rotavirus (severe) cases	0.247	0.247	0.281	Bangladesh and Ghana [32] Malawi [33]

**Mean duration of illness (in days)**				
Rotavirus (non-severe) cases	6	3	6	Assumption
Rotavirus (severe) cases	6	5	6	Assumption

**Cumulative age distribution of disease**				
<3 months:	0.5%	7.1%	6.6%	Bangladesh: [42] Ghana: [43] Malawi: [44]
<6 months:	6.6%	24.2%	26%	
<12 months:	51.1%	70%	77.7%	
<24 months:	97.1%	98.6%	99.5%	
<60 months:	100%	100%	100%	

**Health service costs**				
Government cost per visit				
*Non-severe rotavirus cases*				
Facility (outpatient)	$1.88	$1.61–$6.15*	$7.02–$8.02*	Bangladesh: [45] Ghana: [46] Malawi: [15]
*Severe rotavirus cases*				
Facility (inpatient)	$11.41	$22.71–$48.57*	$8.02–$46.34*	Bangladesh: [47] Ghana: [38] Malawi: [15]

Household cost per visit				
*Non-severe rotavirus cases*				
Informal	$1.17	–	–	Bangladesh: [37]
Facility (outpatient)	$1.39	$0.72–$2.75*	$0.09–$5.80*	Bangladesh: [37] Ghana: calculated using [48] Malawi: [15]
*Severe rotavirus cases*				
Informal	$1.17	–	–	Bangladesh: [37]
Facility (inpatient)	$51.21	$10.17–$21.74*	$0.11–$13.75*	Bangladesh: [39] Ghana: calculated using [37] Malawi: [15]

* Ranges reflect costs associated with care at different facility types.

In each analysis, the model tracks the designated birth cohorts over the first five years of life. Individuals may or may not get disease. If they get rotavirus disease, it can be severe or non-severe, and treatment may or may not be received and can occur at informal (Bangladesh only), outpatient, or inpatient facilities. Non-severe disease results in recovery, whereas severe disease may result in recovery or death.

### 2.2 Alternative data inputs to reflect new vaccine products

Data inputs altered to account for alternative vaccine products include the number of doses, coverage of the third dose (if applicable), vaccine wastage, efficacy, incremental health system cost, and vaccine price (Table 2).

**Table 2:** Model inputs that vary by vaccine selection.

	Bangladesh (Rotarix®; ROTAVAC®; ROTASIIL®)	Ghana (Rotarix®; ROTAVAC®; ROTASIIL®)	Malawi (Rotarix®; ROTAVAC®; ROTASIIL®)	Sources
Doses	2; 3; 3	2; 3; 3	2; 3; 3	[11]

*Vaccine coverage in year of introduction*				
Dose 1	97%	94%	90%	Bangladesh: [49] Ghana: [50] Malawi: [36]
Dose 2	96%	92%	87%	
Dose 3	94%	90%	84%	
Vaccine wastage	5%; 25%; 5%	5%; 25%; 5%	5%; 25%; 5%	[11], [18]

Vaccine efficacy, severe disease*				
Full course	48%	65%	64%	Bangladesh: [51] Ghana: [52] Malawi: [36]
Waning (relative decrease per year)	36.0%	54.7%	47.5%	
Incremental health system cost per dose	$0.54	$1.30	$0.42	Bangladesh: derived from [53] Ghana: [54] Malawi: derived from [55]

* Effectiveness data was used in Malawi though we use the term efficacy for consistency across countries.

Rotarix® has a two-dose schedule, while ROTAVAC® and ROTASIIL® have three-dose schedules [11]. We incorporate the third dose for ROTAVAC® and ROTASIIL® with coverage at DTP3 levels. Rotarix® is modelled as a single-dose presentation with 5% wastage [11]. ROTAVAC® is modelled as a five-dose presentation, resulting in smaller storage volume but a higher wastage rate of 25% [17], [18]. Some sources suggest wastage as high as 50% for this presentation, but we utilized a lower wastage to reflect the 5-dose presentation and expectations that wastage may fall over time [11]. ROTASIIL® is modelled as a two-dose presentation and a larger volume than Rotarix® and ROTAVAC®. When examining ROTASIIL®, we maintain the 5% wastage rates utilized in our prior country analyses of Rotarix® [11]. While vaccine efficacy may differ by product, we do not believe there is sufficient evidence to differentiate by product in these countries, so we assume values consistent with the prior analyses [19]. Incremental health system cost per dose depends on various factors such as vaccine presentation, vaccine volume, cold chain and transport requirements, delivery process, and training needs, but quantifying these components precisely is challenging without extensive in-country data collection. As data collection is beyond the scope of this analysis, we conducted a threshold analysis to examine how changes in incremental health system costs influence the results.

When comparing alternative vaccine products, we consider two alternative price scenarios in each country (Fig. S1 in supplementary materials). The first scenario assumes the country pays the Gavi price, which would remain constant for all years in the analysis. For this scenario we used a Gavi price-per-dose of US$2.02, $2.00, and $1.00 for Rotarix®, ROTASIIL® and ROTAVAC®, respectively [11], [20]. The second scenario assumes that each country would support a co-financing share per dose based on Gavi’s co-financing policy and each country’s co-financing status [8], [21]. Malawi is an Initial Self-Financing country supporting a minimum co-financing of $0.20 per dose of vaccine. Per Gavi’s policy, a country in this group selecting a three-dose rotavirus vaccine pays only two-thirds of the per-dose price, so a three-dose rotavirus vaccine represents the same co-financing commitment as a two-dose vaccine [22]. Malawi is assumed to remain in this phase for the period of analysis. Bangladesh is a Preparatory Transition country, meaning that its co-financing share would increase by 15% per year. We assume Bangladesh stays in this phase over the duration of the analysis. The average cost per dose for Bangladesh is therefore $0.14, $0.29, and $0.29 for ROTAVAC®, ROTASIIL®, and Rotarix®, respectively. Ghana entered the Accelerated Transition phase in 2017; assuming Ghana remains in this phase, the co-financing share increases linearly until full price is reached in 2022^1^. The average co-financing per dose over the period of analysis for Ghana is $0.78, $1.55, and $1.57 for ROTAVAC®, ROTASIIL®, and Rotarix®, respectively.

In addition to examining a variety of scenarios, we also undertook a threshold analysis to examine the robustness of our results by varying the incremental health system cost per dose of the alternative products. Specifically, we reduced the incremental health system cost per dose for more costly products until the total cost of delivery and cost-effectiveness ratio were equivalent to the values obtained for the lowest cost product. The incremental health system cost per dose includes all non-vaccine-related costs such as vaccine distribution, cold chain expansion, staff training, and labor for service delivery or program management.

## 3 Results

The principal results of this study are health impact, cost-effectiveness, and cost comparisons among three rotavirus vaccine products for three countries at different phases of Gavi support. Health benefits provided by rotavirus vaccination remain unchanged from prior studies and are similar across vaccines in the same country as we assume comparable protection conferred by different products. Detailed impact results are available in Table 3.

**Table 3:** Key model outputs by country.

	Bangladesh	Ghana	Malawi
Baseline year	2017	2012	2013
Vaccinated cohorts	10	20	20
Baseline rotavirus admissions (annual)	160,000	22,000	15,000
Baseline rotavirus cases (annual)	1.5 million	390,000	290,000

Model output with vaccination over period of analysis, benefits discounted*			
Deaths averted per 100,000	129	1047	662
DALYs averted per 100,000	4230	29,872	20,918
Cases averted per 100,000	126,271	261,574	157,460
Inpatient visits averted per 100,000	14,707	14,857	11,996
Outpatient visits averted per 100,000	39,862	109,108	84,384
Informal “visits” averted per 100,000	56,656	n/a	n/a

* For ease of reading, figures have been adjusted by 100,000 live births, absolute figures available in Table S3 in supplementary materials.

The discounted cost per DALY averted compared to no vaccination ranged from 0.3 to 1.3 times GNI per capita for all vaccines. With the Gavi subsidy, the average cost-effectiveness ratios were below 0.3 times GNI per capita in all three countries. Cost and cost-effectiveness results for the three countries and the three vaccines are displayed in Table 4. Within an individual country, all vaccines had generally similar cost-effectiveness ratios compared to no vaccination. However, in most cases the two-dose vaccine Rotarix® was the most cost-effective followed by ROTAVAC® and then ROTASIIL®.

**Table 4:** Cost and cost-effectiveness by country and vaccine product.

	Bangladesh			Ghana			Malawi		
	Rotarix®	ROTAVAC®	ROTASIIL®	Rotarix®	ROTAVAC®	ROTASIIL®	Rotarix®	ROTAVAC®	ROTASIIL®
*Model output with vaccination over period of analysis, benefits and costs discounted (millions)*									
Health costs averted (government/societal)	$7.0/$33.7			$6.3/$9.1			$8.0/$9.2		
Total cost of vaccination program with/without Gavi subsidy	$41.6/$135.7	$53.5/$141.4	$61.7/$200.5	$67.9/$88.9	$81.5/$101.0	$100.5/$131.3	$10.2/$42.1	$14.5/$43.3	$13.5/$62.6
Cost of vaccine with/without Gavi subsidy	$15.1/$109.3	$14.1/$102.1	$22.3/$161.1	$35.2/$56.2	$32.8/$52.4	$51.9/$82.7	$3.5/$35.5	$4.5/$33.3	$3.5/$52.6
Incremental health system cost	$26.4	$39.4	$39.4	$32.7	$48.6	$48.6	$6.7	$10.0	$10.0

*Model output with vaccination over period of analysis, benefits and costs discounted*									
Cost/DALY averted with/without Gavi subsidy	$61/$789	$153/$833	$216/$1290	$230/$312	$283/$360	$358/$479	$7/$241	$38/$250	$32/$392
Cost/DALY averted as a share of GNI per capita with/without Gavi subsidy	0.05/0.59	0.12/0.63	0.16/0.97	0.16/0.23	0.21/0.26	0.26/0.35	0.02/0.75	0.12/0.78	0.10/1.23
GNI per capita, Atlas method (2016)*	$1330			$1380			$320		

* https://data.worldbank.org/indicator/NY.GNP.PCAP.CD.

Co-financing, which varies by Gavi status, is critical to understanding the costs of the vaccination programs. As such, we examine these costs by country at different stages of Gavi support. Note that overall costs are not comparable across countries due to differing population sizes, birth cohorts, and other factors.

### 3.1 Initial self-financing

In Malawi, the total cost of the vaccine program with a Gavi subsidy ranged from $10.2 million using Rotarix® to $14.5 million using ROTAVAC®, and from $42.1 million using Rotarix® to $62.6 million using ROTASIIL® without a subsidy. The total cost of the program to the country, accounting for the Gavi subsidy, was lowest using Rotarix® followed by ROTASIIL® and then ROTAVAC®. The total cost of the program without accounting for the subsidy, thus representing the joint country and Gavi cost, was again lowest using Rotarix® then followed by ROTAVAC® and ROTASIIL®. Note the ordering by vaccine is different from the country and Gavi perspectives. If we only consider the cost of the vaccine from the country perspective, Rotarix® and ROTASIIL® were equally costly and the cost of ROTAVAC® was higher. ROTAVAC®, however, was the least costly vaccine when considering the country and Gavi vaccine cost together. Rotarix® vaccine costs were only slightly higher followed by higher vaccine costs for ROTASIIL®. Incremental health system costs borne by countries include everything but the vaccine costs and procurement charges (e.g., international shipping and handling, wastage). Two-dose regimens were therefore two-thirds the cost of three-dose regimen.

### 3.2 Preparatory transition

In Bangladesh, the total cost of the vaccination program with a Gavi subsidy ranged from approximately $42 million using Rotarix® to nearly $62 million using ROTASIIL®, and from almost $136 million using Rotarix® to approximately $200 million using ROTASIIL® without a subsidy. The total cost of the program, accounting for the Gavi subsidy and thus representing the cost to the country, was lowest using Rotarix® followed by ROTAVAC® and then ROTASIIL®. The total cost of the program without accounting for the Gavi subsidy followed the same order. Considering only the cost of the vaccine from the country perspective, ROTAVAC® was the least expensive followed closely by Rotarix® and then ROTASIIL®. The same ordering held when considering the country and Gavi vaccine cost together. Incremental health system costs for the two-dose course were again two-thirds of the three-dose course.

### 3.3 Accelerated transition

In Ghana, the total cost of the vaccination program with a Gavi subsidy ranged from approximately $68 million using Rotarix® to $100.5 million using ROTASIIL® and from nearly $90 million using Rotarix® to approximately $130 million using ROTASIIL® without a subsidy. The total cost of the program to the country, accounting for the Gavi subsidy, was lowest using Rotarix® followed by ROTAVAC® and then ROTASIIL®. The total cost of the program, without accounting for the Gavi subsidy, followed the same order. Considering the cost of the vaccine from the country perspective, ROTAVAC® was the least expensive followed closely by Rotarix® and then ROTASIIL®. The same ordering held when considering the country and Gavi vaccine costs together. Incremental health system costs for the two-dose course were again two-thirds of the three-dose course.

**Fig. 1:**
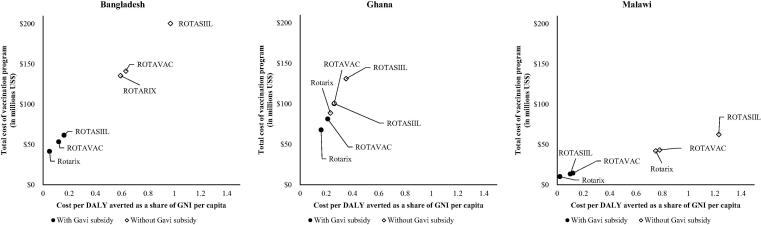
Cost per DALY averted and vaccination program cost.

Fig. 1 shows the cost per DALY averted and total cost of vaccination program for each country and each vaccine, with or without Gavi subsidy. Rotarix® is the least costly and most cost effective product in all scenarios. In the left panel showing Bangladesh, the location of the vaccines is driven more by the inclusion of a Gavi subsidy or not rather than differences between vaccines. This reflects Bangladesh’s large population and an intermediate level of co-financing support from Gavi. As Gavi support declines, the vaccine program will become more expensive and less cost effective. Ghana already pays a larger share of its vaccine costs so variation in the central panel shows a smaller change in cost and cost effectiveness for any of the vaccines as Gavi support declines. Finally, Malawi benefits from more Gavi support and has a smaller population so the cost of the vaccination program does not reach the same levels as Gavi support declines. However, there is variation in the cost effectiveness of the vaccine program.

### 3.4 Threshold analysis

While Rotarix® resulted in the least expensive vaccination program across the examined countries, we undertook a threshold analysis to understand the consistency of this finding by varying the incremental health system cost per dose of the alternative products. Table 5 illustrates the per-dose incremental health system cost as well as the percentage and absolute reduction in incremental health system costs at which costs and cost-effectiveness are equivalent to Rotarix®. Any incremental health system cost value below the value in the table would result in a cost and cost-effectiveness advantage for the associated vaccine product. For example in Bangladesh with a Gavi subsidy, the incremental health system cost per dose of ROTAVAC® would have to be $0.16 (or 30%) lower than those of Rotarix® for the costs and cost-effectiveness of ROTAVAC® to be equal to Rotarix®. The declines in incremental health system costs varies by product and scenario but ranges from declines of 14% to more than 100% in some instances.

**Table 5:** Incremental health system cost values, percentage and absolute decrease, at which cost and cost-effectiveness are equivalent across products.

	Bangladesh	Ghana	Malawi
*Incremental health system cost values*			
Rotarix® (comparator)	$0.54	$1.30	$0.42
ROTAVAC® w/Gavi subsidy	$0.38	$0.94	$0.24
ROTAVAC® w/o Gavi subsidy	$0.46	$0.97	$0.36
ROTASIIL® w/Gavi subsidy	$0.26	$0.42	$0.28
ROTASIIL® w/o Gavi subsidy	< 0	$0.16	< 0

*Percentage and absolute decrease to achieve cost parity*			
ROTAVAC® w/Gavi subsidy	−30% (−$0.16)	−28% (−$0.36)	−43% (−$0.18)
ROTAVAC® w/o Gavi subsidy	−15% (−$0.08)	−25% (−$0.33)	−14% (−$0.06)
ROTASIIL® w/Gavi subsidy	−52% (−$0.28)	−68% (−$0.88)	−33% (−$0.14)
ROTASIIL® w/o Gavi subsidy	N/A	−88% (−$1.14)	N/A

## 4 Discussion

Rotavirus vaccination substantially reduces rotavirus illness, hospitalization, and death in each of the examined countries. In addition, each of the vaccine products is projected to be highly cost-effective relative to no vaccination from the country perspective, even with conservative cost-effectiveness thresholds [23], [24]. We did not undertake a head-to-head cost-effectiveness comparison of the vaccines, which would require more detailed vaccine performance and cost of delivery data and would not be appropriate for this analysis. Rather, our results indicate that choosing any of the vaccine products would be highly cost-effective relative to no vaccination [25].

This work examined how costs may vary across vaccine products, countries at different levels of Gavi support, payer, and cost category. Rotarix® resulted in the lowest-cost vaccination program from both the country and country plus Gavi perspective (Fig. 1). There is no single driver of this finding. However, it is clear that the two-dose schedule for Rotarix® plays an important role in determining both vaccine and health systems costs. Interestingly, lower wastage rates (e.g. 5%) for ROTAVAC® had little impact on our findings. While Rotarix® was uniformly less costly and more cost-effective across these countries (assuming equal effectiveness across products), this finding is sensitive to relatively modest changes in input values. Table 5 demonstrates that relatively small decreases in the incremental health system cost per dose for one of the new vaccines has the potential to tilt the economic benefits in favor of a new vaccine. The small magnitude of changes necessary and the general uncertainty around estimates of incremental health system costs per dose suggest that the economic benefits of alternative vaccines should be considered carefully, ideally through a country-specific evaluation. This may not be feasible in all cases, but this analysis suggests additional study would be valuable in some settings.

This analysis has several limitations. We do not have sufficient information to differentiate vaccine effectiveness or waning across products, and we assume all vaccine products have equivalent effectiveness over time. Future studies may help clarify performance differences. This analysis is therefore driven by cost difference: particularly, by the interplay of doses per course, per-dose price, co-financing, and wastage rates. Critically, we do not have precise estimates of incremental health systems cost per dose for current products or yet know how this cost may vary by product. Likewise, estimates of wastage are uncertain for newer products and rates may fall over time as delivery is optimized. To address these concerns, we conducted threshold analyses and indicated the incremental health system cost thresholds necessary for the new vaccines to meet the cost and cost-effectiveness results of Rotarix®. It will be critical to empirically examine the actual delivery costs associated with the new vaccines, especially given the effects of relatively small changes in incremental health system costs. Finally, this analysis does not examine all Gavi countries, but a selection based on prior modelling studies representing the three main transition phases of Gavi support. We believe these results are informative, but because they only reflect a specified time period, broader analyses would likely lead to new learnings. As countries move closer to graduation and beyond, they may potentially lose access to “tail” prices [26]. Given the higher prices that non-Gavi countries currently pay for rotavirus vaccines, an analysis using higher prices for Rotarix® is likely to be more favorable to new vaccine products. As these cost-effectiveness and impact studies are typically done for 10- or 20-year periods, it will be important for countries to consider the longer-term implications of vaccine choices as well as anticipated changes in the cost structure of countries’ immunization programs.

The analysis conducted here supports the continued use of Rotarix® in Gavi-eligible countries. Because each of the examined vaccine products is projected to be highly cost-effective, countries should consider the relative merits of all products.

This analysis does not seek to present a case for choosing one vaccine product over another but rather demonstrates that all examined rotavirus vaccines would have substantial health impact and be highly cost-effective relative to no vaccination. It provides health economic evidence that may help inform vaccine choice alongside a number of other factors. Country-specific analyses would be key to account for parameters driving results such as differences in incremental health system costs linked to any product.

## 5 Conclusion

This analysis demonstrates that all of the examined rotavirus vaccines would be highly cost-effective relative to no vaccination. We found Rotarix® to be the least costly and most cost-effective product in the three countries analyzed, but the differences can be small and subject to change with minor adjustments to uncertain input variables. A similar analysis examining non-Gavi countries paying higher vaccine prices would likely highlight additional economic benefits of the new vaccines.

## Footnotes

1 In 2018, Ghana re-entered the Gavi Preparatory Transition phase, although to ensure consistency with the previous cost-effectiveness study, we do not reflect this change in this analysis.
