# Supplementary material for: Re-evaluating the cost and cost-effectiveness of rotavirus vaccination in Bangladesh, Ghana, and Malawi: A comparison of three rotavirus vaccines
Source: Vaccine. 2018 Nov 26;36(49):7472–8. doi: 10.1016/j.vaccine.2018.10.068 (PMC6238205; doi:10.1016/j.vaccine.2018.10.068)
Supplement: Supplementary data 1 [file mmc1.docx]

**Supplementary materials**

*Table S1. Study characteristics consistent with prior analyses*

|  | **Bangladesh** | **Ghana** | **Malawi** |
| --- | --- | --- | --- |
| **Model** | TRIVAC | TRIVAC | TRIVAC |
| **Vaccine** | Rotarix® | Rotarix® | Rotarix® |
| **Comparator** | No vaccination | No vaccination | No vaccination |
| **Perspective** | Societal | Societal | Societal |
| **Vaccine introduction year** | 2017 | 2012 | 2012 |
| **Number of birth cohorts** | 10 | 20 | 20 |
| **Monetary units** | 2016 USD | 2015 USD | 2014 USD |
| **Discounting** | Costs and benefits, 3% | Costs and benefits, 3% | Costs and benefits, 3% |
| **Outputs** | Deaths, DALYs, cases, visits and health costs averted; cost per DALY averted; cost of vaccination program | Deaths, DALYs, cases, visits and health costs averted; cost per DALY averted; cost of vaccination program | Deaths, DALYs, cases, visits and health costs averted; cost per DALY averted; cost of vaccination program |

*Figure S1. Projection of price and co-financing share per dose*


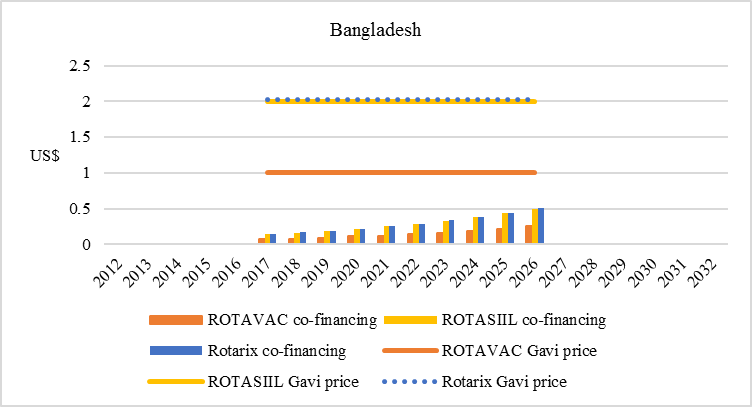

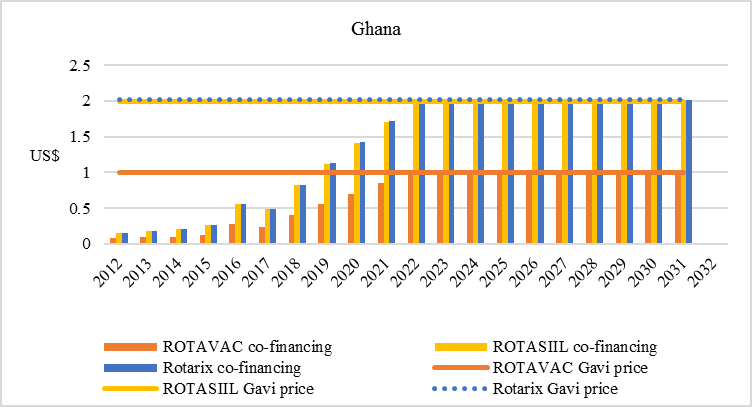

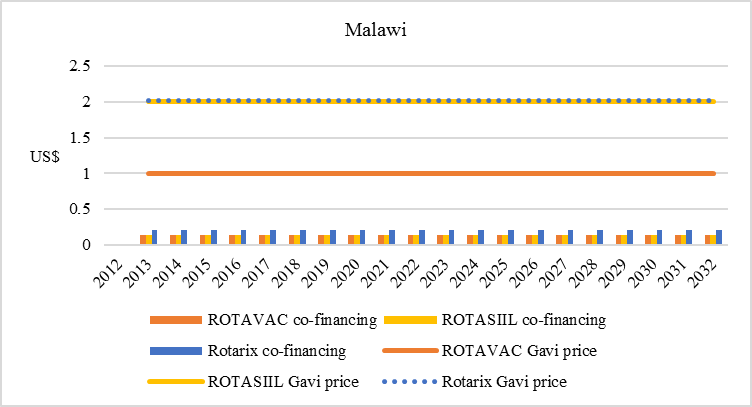


*Table S2. Key model outputs by country – Absolute numbers*

|  | **Bangladesh** | **Ghana** | **Malawi** |
| --- | --- | --- | --- |
| Baseline year | 2017 | 2012 | 2013 |
| Vaccinated cohorts | 10 | 20 | 20 |
| Baseline rotavirus admissions (annual) | 160,000 | 22,000 | 15,000 |
| Baseline rotavirus cases (annual) | 1.5 million | 390,000 | 290,000 |
|  | | | |
| **Model output with vaccination over period of analysis, benefits discounted** | | | |
| Deaths averted | 3,937 | 8,946 | 4,314 |
| DALYs averted | 129,289 | 255,142 | 136,319 |
| Cases averted | 3,859,734 | 2,234,147 | 1,026,141 |
| Inpatient visits averted | 449,542 | 126,900 | 78,179 |
| Outpatient visits averted | 1,218,469 | 931,910 | 549,919 |
| Informal “visits” averted | 1,731,820 | n/a | n/a |

*Table S3. Least costly vaccine product by country and cost category*

|  | **Bangladesh** | **Ghana** | **Malawi** |
| --- | --- | --- | --- |
| Total cost of vaccine program | Rotarix® | Rotarix® | Rotarix® |
| Total cost of vaccine program to country | Rotarix® | Rotarix® | Rotarix® |
| Total vaccine cost | ROTAVAC® | ROTAVAC® | ROTAVAC® |
| Total vaccine cost to country | ROTAVAC® | ROTAVAC® | Rotarix®/ ROTASIIL® |
| Incremental health system cost | Rotarix® | Rotarix® | Rotarix® |
